# A Novel Alkyd-Based Composite Modification System for Achieving High-Performance Acrylic Coatings on Bamboo

**DOI:** 10.3390/polym17081051

**Published:** 2025-04-13

**Authors:** Xuening Gao, Jianfei Zhu, Yuan Zhu, Chengxin Xie, Xianzhang Wu, Xiangchao Pang, Wang Wang

**Affiliations:** 1 State Key Laboratory of Utilization of Woody Oil Resource, Central South University of Forestry and Technology, Changsha 410004, China; gaoxuening2022@163.com (X.G.);; 2 College of Materials Science and Engineering, Central South University of Forestry and Technology, Changsha 410004, China; 3 State Key Laboratory of Efficient Production of Forest Resources & MOE Key Laboratory of Wooden Material Science and Application, Beijing Forestry University, Beijing 100083, China

**Keywords:** acrylic resin, alkyd resin, modification, adhesion, toughness, coating

## Abstract

Acrylic resins are widely favored for bamboo protective coatings due to their superior weather resistance; however, their widespread application is limited by their inherent drawbacks, including brittleness, inadequate adhesion, and poor water resistance. In this study, an innovative composite modification strategy, pre-blending alkyd resin with selected modifiers, was developed to enhance the adhesion, water resistance, and toughness of acrylic resin paint films. Compared to unmodified acrylic resin, the optimal group exhibited enhanced adhesion strengths of 4.21 MPa on tinplate and 7.36 MPa on bamboo, representing improvements of 31.56% and 29.35%, respectively. This was accompanied by a 205 g increase in scratch resistance and a 44% decrease in water absorption, indicating a concurrent enhancement in toughness, strength, and water resistance within the composite system. As revealed by X-ray photoelectron spectroscopy (XPS), differential scanning calorimetry (DSC), and Fourier transform infrared spectroscopy (FTIR) analyses, this enhancement was attributed to the formation of a multidimensional network structure arising from synergistic interactions among the modifier, the alkyd resin, and the acrylic resin. This study provides a theoretical basis for developing high-performance coatings for bamboo protection.

## 1. Introduction

Bamboo, increasingly recognized as a sustainable alternative to timber, holds significant promise for structural and decorative applications [1,2,3]. However, the inherent susceptibility of bamboo to weathering necessitates the development of durable protective coatings to ensure its longevity and maintain its esthetic appeal [4]. While acrylic resins offer desirable attributes such as excellent weather resistance, their widespread adoption in bamboo coatings is limited by their inherent deficiencies in mechanical toughness, interfacial adhesion, and barrier properties, particularly water resistance [5,6,7]. The scientific challenge, therefore, resides in overcoming these limitations to engineer high-performance acrylic coatings specifically tailored for bamboo, which is in high demand.

Numerous studies have focused on enhancing waterborne acrylic resin coatings, including modifications with epoxy, organic fluorine, polyurethane (PU), alkyd, and cross-linked monomers [8,9,10,11]. Gamma-aminopropyltriethoxysilane (KH550) has been shown to enhance mechanical properties by promoting cross-linking within acrylic resin and forming a silane molecular layer at the substrate interface [12,13,14]. Previous studies have demonstrated that integrating waterborne PU with acrylic resin emulsions enhances hardness and thermal stability compared with using acrylic resins alone, key enhancements for achieving the high-performance protective coatings required for durable bamboo products [15,16]. Similarly, hexamethoxymethyl melamine (HMMM) is widely used as a cross-linking agent in resin formulations [17,18,19], while nano-silica has been explored as a filler to improve the tribological and mechanical properties of acrylic coatings [20,21]. Despite improvements in mechanical properties achieved through network formation or physical filling, a key limitation of many modified acrylic resins remains their inherent brittleness. This compromises their long-term durability in outdoor environments, where lignocellulosic materials require coatings that exhibit both high strength and toughness to withstand external stresses [22,23].

Alkyd resins, synthesized through the esterification of renewable polyols, polyacids, and vegetable oils or their derivatives, are recognized for their exceptional flexibility, adhesion, mechanical properties, and inherent weather resistance [24,25]. The presence of unsaturated double bonds enables alkyd resins to undergo self-oxidation and cross-linking in ambient conditions, facilitating efficient film formation without the need for external heat or catalysts. Alkyd resins have shown promise as modifiers in polyurethane and epoxy systems, enhancing water resistance, adhesion, and toughness [26,27]. Their incorporation offers two key advantages: promoting cross-linking through auto-oxidative reactions and increasing toughness and hydrophobicity via long aliphatic chains. However, the inherent auto-oxidative cross-linking of alkyd resins alone often proves insufficient to achieve the high cross-linking density required for robust acrylic-based coatings, particularly for demanding bamboo applications. Therefore, we hypothesized that the strategic integration of alkyd resins alongside a selected suite of modifying agents would promote a concurrent improvement in the mechanical strength, toughness, and water resistance properties of acrylic resin paint films for bamboo substrates.

In this study, alkyd resin was blended with a suite of additives (KH550, HMMM, polyurethane, and nano-silica), and then incorporated into an acrylic resin emulsion. The resulting emulsion was applied to tinplate and bamboo samples, and the properties of the modified paint films were investigated. The enchantment mechanism was elucidated using Fourier transform infrared (FTIR) spectroscopy, scanning electron microscopy (SEM), and X-ray photoelectron spectroscopy (XPS). This composite modification strategy, employing alkyd resins and modifiers, significantly enhanced key performance attributes, notably surface protection, in acrylic coatings for bamboo applications. In addition, it offers valuable perspectives for future research aimed at optimizing these modified acrylic coatings.

## 2. Experimental Materials and Methods

### 2.1. Materials

Waterborne acrylic resin (CAP-200, with a solid content of 46%), waterborne alkyd resin (SA–700, with a solid content of 50%), and waterborne single-component polyurethane (1688, with a solid content of 30%) were purchased from Shanghai Showa Polymer Co., LTD. (Shanghai, China). HMMM was obtained from Jining Juyue New Materials Co., LTD. (Jining, China). KH550 and nano-silica (99.5%, 15 ± 5 nm) were purchased from Shanghai Merrill Biochemistry Co., LTD. (Shanghai, China). Dipropylene glycol butyl ether (DPNB) (98%, AR) was purchased from Shanghai Sinopharm Chemical Reagent Co., LTD (Shanghai, China). Tinplate was obtained from Baoshan Iron and Steel Co., LTD (Guangzhou, China). Bamboo strips were machined from 5-year-old *Phyllostachys edulis* (Moso bamboo), sourced from Hunan Taohuajiang Bamboo Technology Co. (Yiyang, China). All chemicals were used as received without further purification unless otherwise specified. Deionized (DI) water (18.2 MΩ·cm) was used.

### 2.2. Preparation of Modified Acrylic Resin Emulsion

Acrylic resin, dipropylene glycol n-butyl ether (DPNB), modifiers (HMMM, KH550, polyurethane, and nano-silica), and alkyd resin were sequentially added according to Table 1. DI water was then introduced to bring the total volume to 100%, with continuous stirring at 500 rpm for 60 min. The composite emulsions and films of acrylic resin mixed with different modifiers (KH550, HMMM, nano-silica, PU) were designated as AA-KH, AA-HM, AA-SiO_2_, and AA-PU, respectively. Similarly, the composite emulsions and films containing acrylic resin, alkyd resin, and the modifiers were labeled AA-AC, AA-AC-KH, AA-AC-HM, AA-AC-SiO_2_, and AA-AC-PU, respectively. The compositions of the modified acrylic resin emulsions are summarized in Table 1.

The prepared modified acrylate emulsions were stored at 25 °C for 6 months. Their visual appearance was periodically observed and photographed to evaluate their stability, monitoring for delamination, precipitation, and agglomeration. The particle size of the emulsion was measured using a nanoparticle sizer (ZEN1690, Malvern Instruments Ltd., Malvern, UK). The zeta potential of the modified resin emulsions was measured using a zeta potential analyzer (Zetasizer Nano ZS, Malvern Instruments Ltd., Malvern, UK). To ensure the repeatability of the results, each sample was measured three times.

### 2.3. Sample Preparation and Characterization

The paint film was fabricated by applying the prepared coating emulsion onto tinplate and bamboo strips with an automated applicator. The paint film thickness was 100 μm.

The water-based acrylic resins, either modified or unmodified, were poured into a polytetrafluoroethylene (PTFE) mold and allowed to cure at room temperature for 6 h. The pre-dried casting film was heat-treated at 80 °C for another 6 h to complete the curing process before demolding.

The gloss and adhesion of the paint films were evaluated using a mirror gloss meter (BGD-516, Biuged Laboratory Instruments Co., Ltd., Guangzhou, China) and a digital display pull-out adhesion tester (BGD 500, Biuged Laboratory Instruments Co., Ltd., Guangzhou, China), respectively. The scratch resistance of the films was evaluated using an automatic scratch tester (BGD 520, Biuged Laboratory Instruments Co., Ltd., Guangzhou, China) [28]. The surface roughness of the paint films was measured using a portable roughness meter (Leister IR200, Marh Company, Darmstadt, Germany). To ensure the accuracy of the experimental results, each test was performed at least six times to obtain an average value.

The contact angle of the paint film was measured using a contact angle meter (JGW-360B, Chenghui Testing Machine, Zhengzhou, China). A 2 μL water droplet was placed on the surface of the samples, and the contact angle was recorded after a 5 s waiting period. The water resistance of the paint films was evaluated by determining the water absorption rate. The casting films were immersed in DI water, and after 24 h, the specimens were collected and then wiped to remove surface water before weighing.

The formula for calculating water absorption is as follows:

(1) $$H=\frac{M_{1}–M_{0}}{M0}\times 100\%$$

where *H* represents the water absorption of the casting film (%), *M*_0_ denotes the mass of the casting film after drying (g), and *M*_1_ represents the mass of the casting film after the absorption of water (g).

### 2.4. Thermal and Chemical Analysis

A simultaneous thermal analyzer (TG-DSC, PE-STA8000, PerkinElmer, Waltham, MA, USA) was utilized to assess the thermal stability of the paint films. The film samples (diameter of 0.5–3 mm, thickness of less than 2 mm) were transferred to an aluminum crucible, and the samples were subjected to a temperature ramp from ambient to 600 °C within a nitrogen environment, with a heating ramp rate of 10 °C/min.

The chemistry of the acrylic paint film was analyzed using an FTIR spectrometer (Nicolet iS5, Thermo Fisher, Waltham, MA, USA) and an X-ray Photoelectron Spectroscope (K-Alpha, Thermo Scientific, Waltham, MA, USA). The FTIR analysis was performed using the attenuated total reflection mode within a wavenumber range of 4000 to 400 cm^–1^, with 32 scans. The XPS survey spectra were collected in the energy range of 0–1350 eV in 1 eV step, with the detailed peak analysis recorded in 0.1 eV steps. Peak fitting of the XPS C1s spectrum was conducted using Avantage (version 2017) software [29]. A scanning electron microscope (SIGAM 300, ZEISS, Oberkochen, Germany) was employed to analyze the microstructure of the paint film. All samples were sputter-coated with gold in a vacuum chamber for 2–3 min before SEM analysis.

### 2.5. Gel Content Test

The casting films, measuring 50 × 20 × 2 mm^3^, were immersed in acetone for 12 h at room temperature, then dried in an oven at 80 °C for 6 h before weighing for the gel content test. For each group, three parallel specimens were measured, and the average value was taken.

The gel content (*S*) was calculated using the following formula:

(2) $$S=\frac{M_{3}}{M_{2}}\times 100\%$$

where *S* represents the gel content of the casting film (%), *M*_2_ denotes the oven-dried weight of the casting film before acetone immersion (g), and *M*_3_ denotes the oven-dried weight of the casting film after acetone immersion (g).

### 2.6. X-Ray Diffraction (XRD)

The crystalline structure of the casting film was studied using an X-ray diffractometer (Rigaku, Smart Lab SE, Tokyo, Japan). The film samples (diameter of 10–20 mm, thickness of less than 10 mm) were directly scanned at 2θ angles between 3° and 80° with X-rays of 1.5406 Å generated by a CuK_α_ source.

## 3. Results and Discussion

### 3.1. Stability of Modified AA Emulsions 

**Figure 1:**
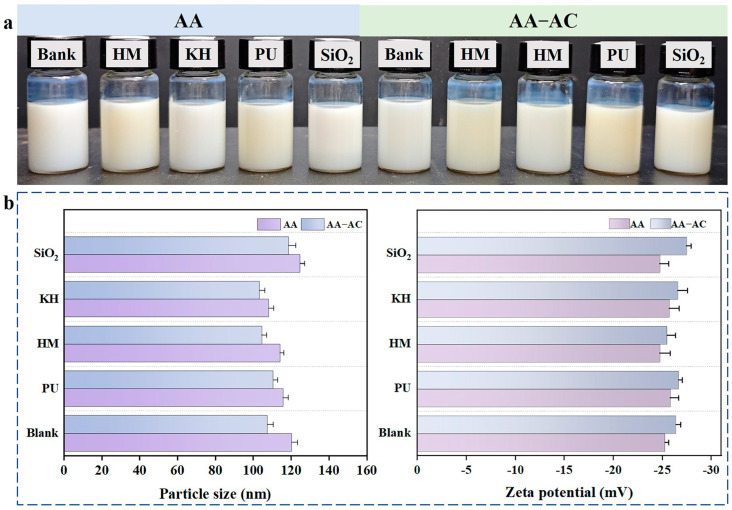
(**a**) The appearance of the modified resin emulsion after storage for 6 months at room temperature; (**b**) the particle size and zeta potential of the modified acrylic resin emulsions after storage for 1 week.

After 6 months of storage, the emulsions in all groups showed no evidence of phase separation, precipitation, or solidification, which indicated that the modifiers (KH550, HMMM, nano-silica, PU, and AC) had no negative impact on the long-term stability of the acrylic resin emulsion (Figure 1a). The particle size and zeta potential of the modified acrylic resin emulsions are shown in Figure 1b. The particle size of the composite AA emulsion decreased with the addition of KH550, HMMM, and PU, which was likely attributed to the averaging effect of the smaller particles of the modifiers (Figure 1b), and this decrease was further enhanced by the introduction of AC. The decreased particle size suggested that the AA emulsification system was not affected by the alkyd resin and other modifiers, which further demonstrated the high stability of the composite system. The zeta potential of a resin emulsion is an important indicator of its stability [30]. The zeta potential of the emulsions was analyzed for significant differences, and no significant difference was observed between the groups (*p* < 0.05). The data suggest that modification with alkyd resin, whether alone or in combination with other modifiers, has no negative impact on the stability of the acrylic resin emulsion. This observation demonstrates the excellent compatibility between the components. Consequently, the modified acrylic resin system retains its structural integrity while preserving its potential to enhance performance in bamboo-related applications.

### 3.2. Physical and Mechanical Properties of Paint Film

**Figure 2:**
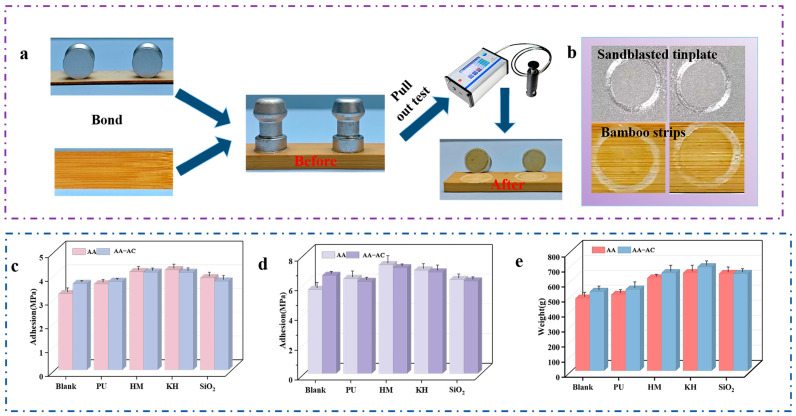
The mechanical properties of the paint films: (**a**) a flow chart for the pull-off adhesion test; (**b**) images of the damaged surface after the adhesion test; (**c**) adhesion on sandblasted tinplate; (**d**) adhesion on bamboo strips; (**e**) the scratch resistance values.

The adhesion of the paint film, which reflects its ability to bond with the substrate, was measured [31]. This index is crucial for assessing the performance of paint films, as it directly influences their durability and service life [32]. Figure 2a presents a flowchart detailing the adhesion testing process, whereas Figure 2b displays the fracture surface after the pull-out adhesion test. Figure 2c,d show the adhesion results of the paint film on sandblasted tinplate and bamboo strips. The unmodified AA exhibited adhesion strengths of 3.20 ± 0.17 MPa and 5.69 ± 0.37 MPa on tinplate and bamboo, respectively. Upon incorporation of the modifier, the adhesion of the resulting paint film increased, with the optimal formulation achieving values of 4.21 ± 0.16 MPa and 7.36 ± 0.35 MPa on tinplate and bamboo, representing enhancements of 31.56% and 29.35%, respectively, relative to the unmodified AA. The enhanced adhesion observed in the AA-KH, AA-HM, and AA-PU groups was probably due to the formation of a network structure between the modifier and the acrylic resin [12,15,17]. The enhanced adhesion of the modified AA-SiO_2_ was attributed to the incorporation of silica nanoparticles as fillers [33]. A previous study has shown that alkyd resins, when mixed with other resins, can improve the flexibility, gloss, and water resistance of paint film [27]. The molecular structure of alkyd resins contains polar groups, such as hydroxyl and carboxyl groups, which enhance the wetting of the coating emulsion and, consequently, improve the adhesion of the paint film. In addition, the alkyd resin chain might be entangled with the acrylic resin chain, thereby forming a network structure and increasing the adhesion of the acrylic resin film. However, after the additional incorporation of alkyd resins, the adhesion of the modified acrylic resins remained essentially unchanged (Figure 2c,d). This was due to the addition of the flexible alkyd resin chains, which may disrupt the cross-linking between the modifier and the acrylic resin, thus counteracting the increased adhesion. In spite of this, the acrylic resin composite system, which included various modifiers and alkyd resins, exhibited excellent adhesion on both tinplate and bamboo strips.

Coatings are often susceptible to external damage, which makes scratch resistance a critical factor in evaluating the applicability of paint films, particularly for outdoor applications [34]. The scratch resistance test results (Figure 2e) indicated that the AA film failed at a load of 485 ± 25 g, while the composite films in groups AA-KH, AA-PU, AA-HM, and AA-SiO_2_ failed at 650 ± 40 g, 505 ± 20 g, 615 ± 15 g, and 645 ± 30 g, respectively. The addition of modifiers enhanced the scratch resistance of the acrylic resin film. This finding is consistent with earlier research demonstrating that modifiers, including KH550, HMMM, and PU, enhance the scratch resistance of paint films by promoting a denser network structure, while nano-silica contributes to this improvement by ensuring the uniform distribution of fillers within the coating. Alkyd resin incorporation enhanced the scratch resistance of the composite system. The optimized AA-AC-KH formulation exhibited a scratch resistance threshold of 690 ± 30 g, representing a 205 g improvement over the unmodified AA group. The flexible chain segments of the alkyd resin help absorb the external force and reduce the stress concentration, which allows the film to better resist the formation and expansion of cracks when subjected to slip injuries. Overall, the combination of alkyd resin with modifiers effectively enhanced and strengthened the acrylic resin paint film, resulting in improved scratch resistance.

In summary, the synergistic combination of alkyd resins and modifiers significantly improved the toughness and strength of the acrylic resin paint film. This enhanced performance, by creating a more robust barrier, provided superior protection against mechanical stress (such as impact and scratching) and environmental factors (like moisture absorption or desorption), thus extending the service life of the coating and the bamboo substrate. Compared to unmodified acrylic coatings, this formulation offered a more effective long-term solution for outdoor bamboo preservation.

**Figure 3:**
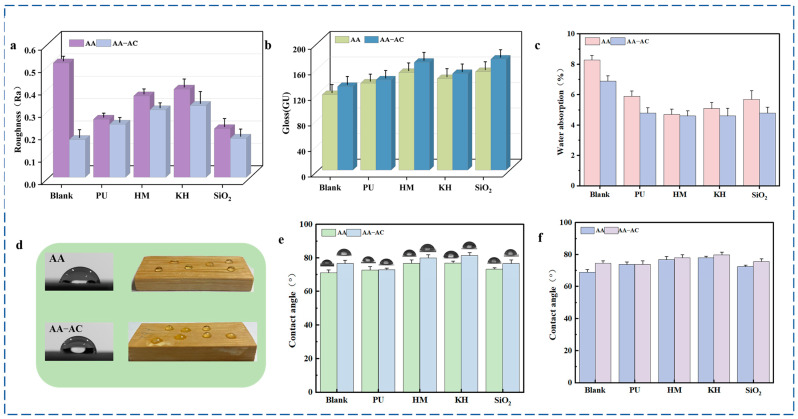
Physical properties of paint films: (**a**) roughness; (**b**) gloss; (**c**) water absorption; (**d**) contact angle test; (**e**) contact angle on tinplate; (**f**) contact angle on bamboo strips.

The surface roughness of the prepared coatings in this study is shown in Figure 3a. Compared with the unmodified AA (0.51 ± 0.021 µm), the coating roughness decreased with the addition of modifiers (nano-silica, KH550, PU, and HMMM), with AA-SiO_2_ exhibiting the lowest roughness at 0.24 ± 0.032 µm. The modifiers (PU, KH550, and HMMM) enhanced the self-leveling properties of the coating, while nano-silica served as a filler to fill in the coating, thereby smoothing the paint film on a macro level and reducing its roughness. Following the addition of alkyd resin, the film’s roughness further decreased, with the AA-AC group measuring 0.17 ± 0.031 µm, which represented a 68% reduction compared with the AA. The excellent leveling properties of the alkyd resin facilitated the flow and self-leveling of the acrylic resin coating during the drying process, which reduced surface defects. As a result, this further decreased the roughness of the paint film in the acrylic resin, modifier, and alkyd resin composite system.

The gloss of the paint film is a crucial metric for assessing its appearance. The gloss data indicated that the addition of most modifiers (HMMM, PU, and nano-silica) improved the film’s gloss compared to AA (118 ± 5.7 GU). Among the modifiers, nano-silica demonstrated the most substantial enhancement, reaching 173 ± 4.6 GU. The gloss of the modified film further increased after the addition of alkyd resin. This enhancement was attributed to the alkyd resin’s excellent flexibility and leveling properties, which, when combined with the acrylic resin and modifiers, resulted in a smoother and more uniform surface. This finding aligns with the roughness results, suggesting that the surface flatness of the acrylic paint film was enhanced following the composite modification of the alkyd resin with the other modifiers. This enhanced gloss not only improved the esthetics of the outdoor bamboo coating, but also significantly enhanced its weatherability, water resistance, scratch resistance, and ease of cleaning, collectively contributing to an extended service life for bamboo products.

Alkyd resins, acting as hydrophobic modifiers, were shown to enhance the hydrophobicity of acrylic resins. To evaluate their water resistance, water contact angle and water absorption tests were conducted on modified acrylic resin paint films. The AA paint film exhibited water absorption of 8.3% on tinplate. The incorporation of modifiers led to a reduction in water absorption (Figure 3e). This decrease was primarily attributed to the enhanced densification of the acrylic resin matrix achieved by KH550, PU, and HMMM via cross-linking. In addition, silica nanoparticles filled microscopic pores within the acrylic resin, reducing surface defects and hydrophilic channels, thus effectively impeding water permeation. The further addition of alkyd resin resulted in a continued reduction in water absorption. Notably, the AA-AC-HM and AA-AC-KH groups demonstrated the lowest water absorption (4.6%), representing a 44% reduction compared to the AA group. This enhanced water resistance was primarily ascribed to the entanglement of alkyd and acrylic resin chains, coupled with the inherent hydrophobicity of the long aliphatic chains within the alkyd resin, further hindering water penetration.

The AA paint film exhibited water contact angles of 71 ± 1.5° and 69 ± 1.6° on tinplate and bamboo substrates, respectively (Figure 3d–f), a difference that was found to be statistically significant (*p* < 0.05) based on our analysis. The incorporation of modifiers increased the contact angles of the modified acrylic resin coatings on both substrates. This enhancement was primarily attributed to the reaction of modifiers (KH550, PU, and HMMM) with hydroxyl or carboxyl groups within the acrylic resin, as well as the physical filling of nano-silica, optimizing paint film densification and reducing surface polarity, thereby increasing hydrophobicity. The alkyd resin further increased the water contact angles on both tinplate and bamboo. This improvement was primarily attributed to the alkyd resin’s long-chain aliphatic structure, which effectively reduced surface energy and thereby enhanced paint film hydrophobicity. In conclusion, these results suggest that composite modification effectively enhances the water resistance of acrylic coatings, thereby improving the durability and service life of bamboo and promoting its sustainable resource utilization.

### 3.3. Thermal Stability

**Figure 4:**
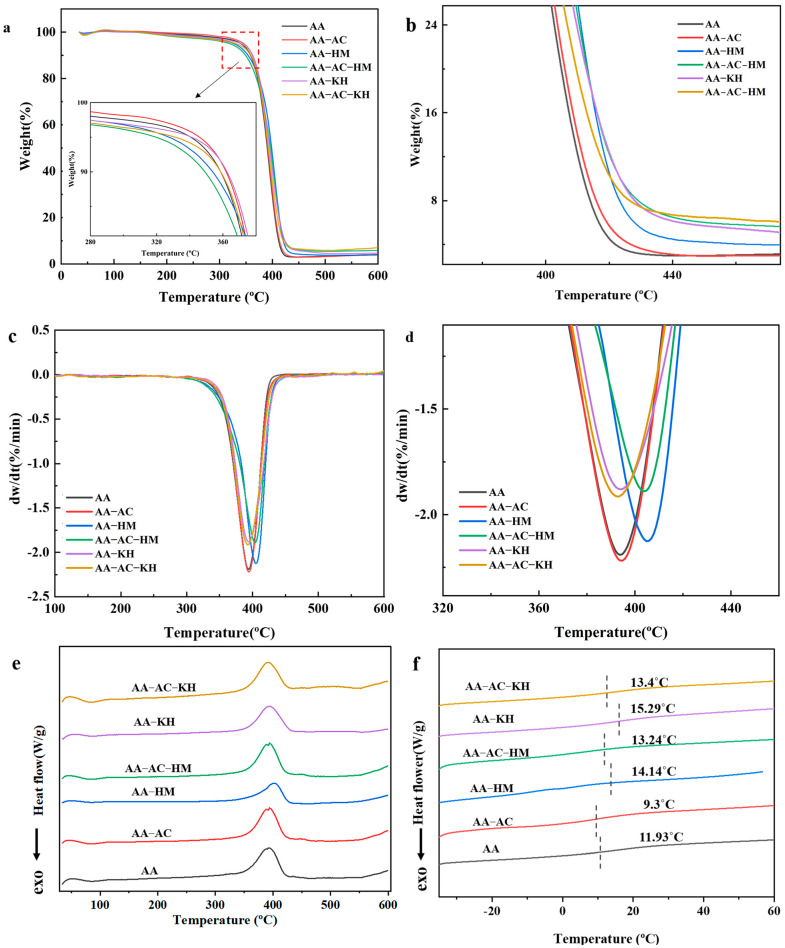
Thermal stability of modified resin paint film: (**a**) TG curves; (**b**) enlarged view of TG curves; (**c**) DTG curves; (**d**) amplified DTG; (**e**) DSC curves; (**f**) Tg curves of acrylic resin composite system.

Thermal stability is a crucial index for evaluating the outdoor durability and service life of paint films. Thermogravimetric (TG) analysis was employed to assess the thermal stability of acrylic resin composite films. The thermal stability curves of the modified resin paint films are presented in Figure 4a, with the extracted data shown in Table 2. Within the temperature range of 30–200 °C, the mass loss observed in resin materials is primarily attributed to the elimination of unbound and bound water [35]. The mass loss occurring between 300 and 450 °C was predominantly due to the thermal decomposition of macromolecular chains [36]. For acrylic composite films, T_onset_, T_max_, and T_end_ were higher than in AA films. The temperature (T_0.5_) at which 50% mass loss occurred was 390 °C for AA and increased to 391 °C and 395 °C for AA-KH and AA-HM, respectively (Table 2). The increase in AA-HM and AA-KH at T_0.5_ was mainly due to the cross-linking of the modifiers (KH550/HMMM) with the acrylic resin to form a network structure, which made the paint film highly thermally stable. With the addition of alkyd resin, the T_onset_, T_max_, and T_end_ of AA-AC, AA-AC-HM, and AA-AC-KH acrylic composite films were higher than those of AA. The unsaturated fatty acid chains in the alkyd resin underwent an autoxidative cross-linking or polymerization reaction, which further enhanced their stability by forming a multidimensional network with the acrylic resin through entanglement.

The Derivative Thermogravimetry (DTG) curves showed a similar weight loss trend, with a peak observed in all the paint films. In the high-temperature range (460–600 °C), the acrylic resin film experienced significantly greater weight loss compared with the modified films. The presence of alkyd resin and HMMM/KH550 further enhanced the thermal stability of the acrylic paint film, as evidenced by the increased residual mass after thermal analysis.

The DSC curves of AA and the acrylic films are shown in Figure 4e. From Figure 4f, it be seen that the Tg of the acrylic composite films with the addition of modifiers (KH550 and HMMM) was higher than that of the AA group (11.93 °C). The acrylic resin formed a cross-linked network with KH550 and HMMM, which led to an increase in the Tg values of AA-KH and AA-HM, respectively. The Tg values of AA-AC, AA-AC-KH, and AA-AC-HM were 9.3, 13.24, and 13.4 °C, respectively. AA-AC-HM and AA-AC-KH composite films showed a decrease in Tg values because the polymer chain segments could move more freely at lower Tg, which improved the flexibility of the material and chain mobility. The decrease in Tg values after the addition of AC indicated improved flexibility of acrylic composite system. The results of the thermal stability tests align with the mechanical property evaluations, confirming that incorporating KH550/HMMM and alkyd resins enhances the overall performance of the acrylic composite film. Consistent with previous studies, these findings demonstrate that the composite modification system enhances the thermal stability of acrylic resin paint films by forming a multidimensional network structure, resulting in reduced thermal degradation.

### 3.4. Characterization of Modified Acrylic Resin Paint Films

**Figure 5:**
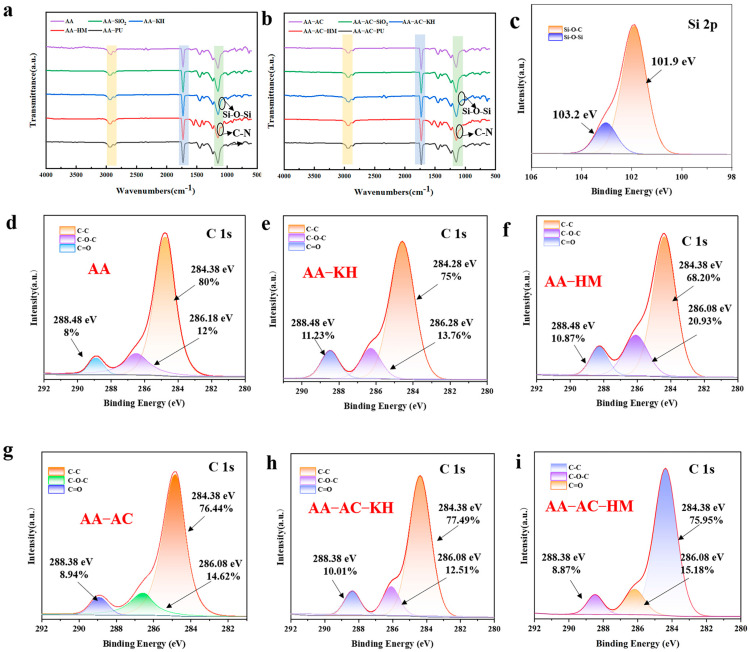
(**a**) FTIR spectra of modified resin; (**b**) FTIR spectra of acrylic resin composite groups; (**c**) high-resolution XPS spectra of Si 2p in the AA-AC-KH system; (**d**–**i**) high-resolution XPS C1s spectra of various acrylic resin film samples.

FTIR characterization was used to assess the changes in alkyd resin-modified acrylic resin before and after modification, as shown in Figure 5a,b. The acrylic resin displayed four characteristic absorption peaks at 3487 cm^–1^, 2945 cm^–1^, 1725 cm^–1^, and 1159 cm^–1^, corresponding to the bending vibrations of the -OH, C-H, C=O, and C-O-C groups, respectively [37]. The absorption peaks of AA exhibited a similar profile to that of AA-AC, suggesting that the addition of AC did not alter the molecular structure of AA. The presence of Si-O-Si and Si-O-C structures was observed with characteristic absorption peaks at 1120 cm^–1^ and 1032 cm^–1^ in the AA-KH spectrum. This result was attributed to the active siloxanyl groups at both ends of KH550, which hydrolyzed into silicone hydroxyl groups and subsequently reacted with the acrylic resin to form Si-O-Si and Si-O-C bonds. The FTIR spectra of AA-HM showed characteristic peaks for ether bonds at approximately 1161 cm^–1^ and 1079 cm^–1^, as well as for the triazine ring at around 1547 cm^–1^. These findings confirmed that the modifiers (AA and KH550) reacted with the AC.

The surface chemistry of the prepared paint films in this study was further analyzed using XPS. As shown in Figure 5d–i, the C1s spectra of the modified resin paint films were deconvoluted into three subpeaks with binding energies at 284.38 eV, 286.18 eV, and 288.48 eV, which corresponded to C-C, C=O, and C-O-C/C-N bonds, respectively. In the C1s peak of AA, the total peak area consisted of 80% C-C, 12% C=O, and 8% C-O-C bonds. Compared to AA, the addition of HMMM and KH550 led to a decrease in C-C bonds and an increase in the ratios of C=O and C-O-C bonds (Figure 5e,f). In the case of KH550, this reduction was attributed to the esterification reaction between the ethoxy group in KH550 and the hydroxyl group in the acrylic resin, which resulted in the cleavage of the original C-C bond and the formation of a new C-O-C bond. The peak fitting of the Si 2p spectrum for AA-AC-KH (Figure 5c) confirmed the formation of Si-O-Si and Si-O-C bonds [38], which further supported the reaction between the acrylic resin and KH550. In the case of HMMM, the increase in C-O-C/C-N bonds was attributed to the reaction between the amino group of HMMM and the carboxyl group in the acrylic resin, resulting in the formation of an amide bond. Concurrently, the C-O bond content increased as the methoxy group in HMMM participated in esterification, binding to hydroxyl groups in the acrylic resin. After the addition of alkyd resin, the percentage of C-C bonds in AA-AC decreased to 76.44%, while the content of C-O-C and C=O bonds increased to 8.94% and 14.62%, respectively (Figure 5g). The unsaturated double bonds (C=C) in alkyd resins are easily oxidized during autoxidation, forming peroxides that decompose to produce free radicals. These free radicals can react with the hydroxyl groups in alkyd resins to form new ether bonds (C-O-C), a process that increases the number of C-O-C bonds. The reduction in the number of C-O-C bonds in AA-AC-HM and AA-AC-KH, compared to AA-HM and AA-KH, was attributed to the addition of alkyd resins, which affects the cross-linking reaction of the acrylic resins with the modifiers (KH550 and HMMM), thereby reducing the number of C-O-C bonds. In summary, the data confirm that a cross-linking reaction between the acrylic resin and the modifiers (KH550 and HMMM) occurred, which enhanced the crosslinking of the acrylic resin composite system.

**Figure 6:**
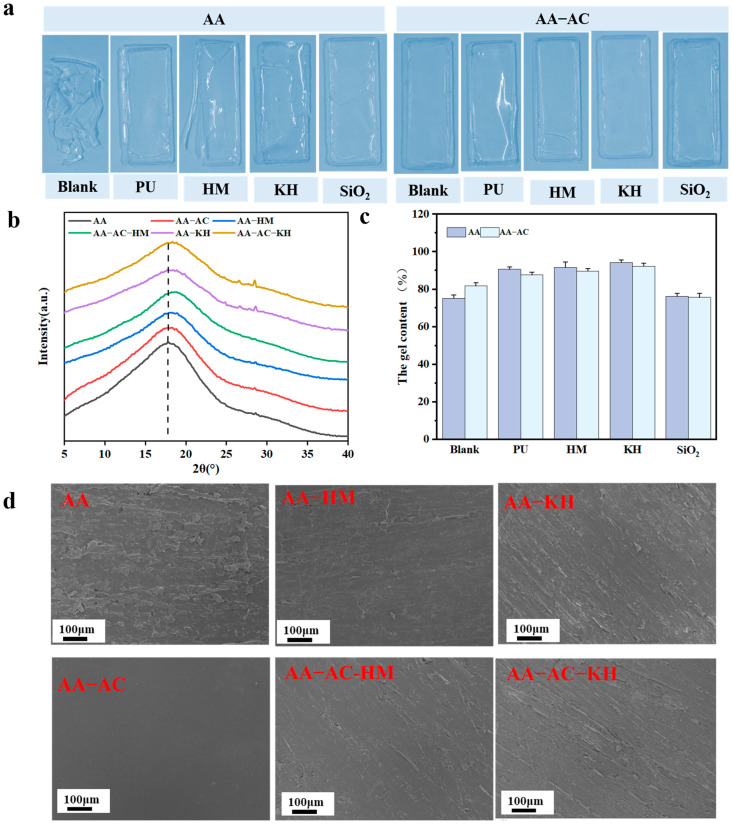
(**a**) Appearance of cured modified resin film; (**b**) XRD patterns of acrylic resin composite system; (**c**) gel content of acrylic resin composite system; (**d**) SEM micrograph of films derived from modified acrylic resin.

As shown in Figure 6a, the unmodified acrylic resin was fragmented, while the modified group (AA-HM, AA-KH, AA-PU, and AA-SiO_2_) possessed a certain degree of integrity; however, it still displayed some cracks. After further addition of alkyd resin, the modified casting films were intact with no cracks on their surfaces. This supports previous results, indicating that the composite modification system significantly enhances the strength and toughness of acrylic resin paint films by creating a multidimensional network structure that improves resistance to external forces. Consequently, when applied to bamboo substrates, it provides more effective substrate protection, reduces cracking and flaking, and extends the substrate’s service life.

As shown in Figure 6b, the decreased area of the peak at 2θ = 17° for the acrylic resin with the addition of modifiers indicated a decrease in the crystallinity of the acrylic resin. The increase in the peak width of AA-HM and AA-KH was mainly due to the cross-linking of the modifier (KH550 and HMMM) with the acrylic resin, which disrupted the original crystalline structure and interfered with the ordering of the acrylic resin’s molecular chains. The increase in the width of the AA-AC peak was mainly because of the physical entanglement between the acrylic resin and the acrylic resin molecular chains. Moreover, the auto-oxidative cross-linking of alkyd resins also destroyed the crystal structure of the acrylic resin. In conclusion, the modifier and the alkyd resin interfered with the crystal structure of the acrylic resin chains, leading to a decrease in crystallinity.

The gel content of the acrylic resin composite film was measured, and the results are shown in Figure 6c. The gel content of the AA group was about 75%, while the gel contents of AA-PU, AA-HM, and AA-KH were 90.7%, 91.55%, and 94.5%, respectively. The cross-linked network between the acrylic resin and the modifier (KH550/PU/HMMM) increased the gel content in the composite system. Following the addition of AC, the gel content of AA-AC increased, whereas that of AA-AC-PU, AA-AC-HM, and AA-AC-KH decreased. The increase in AA-AC gel content was mainly due to autoxidative cross-linking of the alkyd resin and physical entanglement between the alkyd and acrylic resins, which together formed a more stable and dense network structure. The addition of the alkyd resin interfered with the cross-linking of the modifier (PU/HMMM/KH550) with the acrylic resin, thus reducing the gel content of the acrylic resin composite system.

SEM analysis was conducted to further assess the micro-morphology of the paint film surface. The AA group displayed a rough texture with small pores and clusters, likely owing to the high molecular weight of the acrylic resin emulsion, which led to poor leveling performance. In contrast, the film surfaces of the groups modified with HMMM and KH550 showed improved smoothness and increased flatness compared with the AA group. This improvement was attributed to the network structure formed by the modifier (KH550/HMMM) with the acrylic resin, which improved the surface properties of the films. The surface smoothness of the AA-HM and AA-KH paint films was further enhanced by the addition of alkyd resin. This enhancement was attributed to the plasticizing effect of the alkyd resin, which resulted in a smoother and more uniform surface, as evidenced by the comparison between the AA and AA-AC groups.

**Figure 7:**
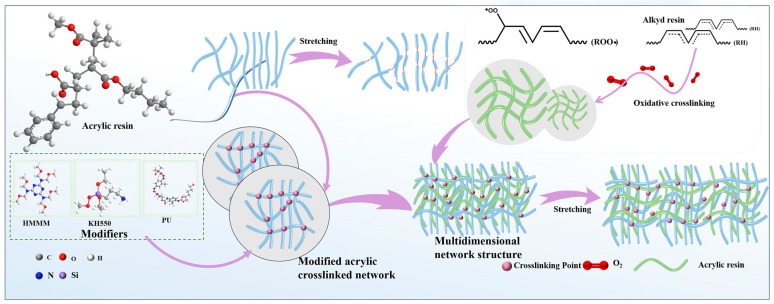
Acrylic resin paint film enhancement and toughening modification mechanism diagram.

Overall, the incorporation of alkyd resins and modifiers enhanced the adhesion, scratch resistance, flexibility, and water resistance of the acrylic resin paint films. According to the characterization results of AA, AA-HM, and AA-KH, along with the findings from AA-AC, AA-AC-HM, and AA-AC-KH, the mechanism by which alkyd resins and modifiers improved the acrylic resin paint film is illustrated in Figure 7. The modifiers mainly enhanced the mechanical properties of the acrylic resin by facilitating the formation of a network structure with it. Oxidative cross-linking of alkyd resins formed a network structure, while the use of alkyd resins as plasticizers improved the mobility of the molecular chains in the resin. Alkyd resins, acrylic resins, and modifiers could form intertwined multidimensional network structures to improve the deformation of acrylic resins against external forces. This improvement enabled the paint film to distribute stress more evenly when subjected to force. Therefore, the composite system composed of acrylic resin, a modifier, and alkyd resin exhibits exceptional mechanical properties and water resistance. This study presents a composite modification strategy employing alkyd resins and modifiers to significantly enhance key performance attributes, including surface protection, in acrylic coatings designed for bamboo applications. The findings offer valuable guidance for designing high-performance acrylic coatings, representing a substantial advancement in bamboo preservation technology by extending product lifespan and promoting sustainable resource utilization.

## 4. Conclusions

In this study, we utilized a composite system of alkyd resins and modifiers to enhance the adhesion, water resistance, and toughness of acrylic resin paint films. The adhesion of the optimal group on tinplate and bamboo was 4.21 and 7.36 MPa, respectively, indicating improvements of 31.56% and 29.35% relative to the unmodified acrylic resin. Additionally, the water resistance and scratch resistance of the paint film were improved. XPS, DSC, XRD, and FTIR analyses showed that these enhancements were attributable to the formation of a unique multidimensional network structure within the acrylic resin matrix, coupled with the plasticizing effect of alkyd resin. These findings not only offer valuable insights for coating design, but also hold significant promise for enabling the wider adoption of bamboo as a sustainable material through the development of high-performance coatings with exceptional durability and protection.

## Figures and Tables

**Table 1:** The mass percentage of each component in the acrylic resin emulsion.

Sample	Acrylic Resin (%)	Alkyd Resin (%)	Modifiers (%) (KH550/HMMM/Nano-Silica/PU)	DPNB (%)
AA	85	0	0	2
AA-HM	85	0	4	2
AA-KH	85	0	4	2
AA-PU	85	0	4	2
AA-SiO_2_	85	0	0.5	2
AA-AC	85	2	0	2
AA-AC-HM	85	2	4	2
AA-AC-KH	85	2	4	2
AA-AC-PU	85	2	4	2
AA-AC-SiO_2_	85	2	0.5	2

**Table 2:** Thermal properties of composite acrylic resin film.

Sample	T_onset_ (°C)	T_max_ (°C)	T_end_ (°C)	T_0.5_ (°C)	Tg (°C)	Residual Mass at 600 °C (%)
AA	368.79	389.82	412.36	390.37	11.93	4.11
AA-AC	370.38	392.81	412.68	391.46	9.3	4.07
AA-HM	376.77	400.77	421.3	391.42	14.14	4.38
AA-AC-HM	369.74	395.59	422.41	398.32	13.24	5.88
AA-KH	370.54	394.18	417.46	395.86	15.29	4.68
AA-AC-KH	368.83	391.81	414.59	395.07	13.4	7.02

## Data Availability

The raw data supporting the conclusions of this article will be made available by the authors on request.
